# Quality Use of Medicines Indicators and Associated Factors in Residential Aged Care Facilities: Baseline Findings from the Pharmacists in RACF Study in Australia

**DOI:** 10.3390/jcm11175189

**Published:** 2022-09-01

**Authors:** Ibrahim Haider, Sam Kosari, Mark Naunton, Theo Niyonsenga, Gregory M. Peterson, Jane Koerner, Rachel Davey

**Affiliations:** 1 Discipline of Pharmacy, Faculty of Health, University of Canberra, Bruce, ACT 2617, Australia; 2 Health Research Institute, Faculty of Health, University of Canberra, Bruce, ACT 2617, Australia; 3 School of Pharmacy and Pharmacology, University of Tasmania, Hobart, TAS 7005, Australia

**Keywords:** potentially inappropriate medications, elderly, quality use of medicines, Beers Criteria, prescribing, residential aged care facilities, factors associated with PIM prescribing

## Abstract

Prescribing potentially inappropriate medications (PIMs), including antipsychotics and benzodiazepines, has been used as an indicator of the quality use of medicines in residential aged care facilities (RACFs). PIMs are associated with an increased risk of falls and hospitalisations in the elderly. The purpose of this study is to assess the extent of prescribing of PIMs in RACFs at baseline in the Pharmacists in residential aged care facilities (PiRACF) study and examine the association of resident and system factors with the number of PIMs. A cross-sectional analysis of 1368 participants from 15 Australian RACFs was performed to detect PIMs using the American Geriatrics Society 2019 Beers^®^ criteria. Most residents (68.1%) were taking at least one regular PIM; 16.9% were taking regular antipsychotics and 11.1% were taking regular benzodiazepines. Long-term proton pump inhibitors were the most frequent class of PIMs. History of falls and higher Charlson Comorbidity Index were associated with an increased number of prescribed PIMs, while dementia diagnosis and older age (85 years or more) were associated with decreased number of PIMs (*p*-value <0.05). Residents in facilities with lower nurse-to-resident ratios were more likely to have an increased number of PIMs (*p* value = 0.001). This study indicates that potentially inappropriate prescribing is common in RACFs and interventions to target residents at highest risk are needed.

## 1. Introduction

People living in residential aged care facilities (RACFs) are at high risk of medication-related harm due to age-associated physiological decline in pharmacokinetic and pharmacodynamic properties [1]. These changes are further complicated by multiple medications, comorbidities, and potential drug–drug and drug–disease interactions [2,3,4].

Quality use of medicines (QUM) refers to the optimal use of medications to maximise the benefit of treatment and limit any medication-related harm [5]. No standardised set of QUM indicators has been widely adopted in RACFs, but prescribing of potentially inappropriate medications (PIMs) [6,7,8,9], antipsychotics [10,11,12,13,14,15], benzodiazepines [16,17,18], and highly anticholinergic medications [19] have all been used as markers for QUM. A PIM refers to a medication for which the risk of adverse events outweighs the clinical benefit [20]. There are different validated tools used to identify PIMs in the elderly, with the most commonly used being the Beers^®^ criteria, developed by the American Geriatrics Society, which is based on systematic reviews of evidence and expert consensus [21]. The presence of PIMs has been associated with significant adverse events, hospitalisations, and death among older people [6,7,8,9]. A recent meta-analysis of 33 studies revealed a statistically significant association between hospitalisation and PIMs [7]. Another meta-analysis of 21 studies found PIMs to be associated with increased odds of hospital admissions and emergency department visits [22]. Amongst PIMs, the use of antipsychotics has been associated with increased risk of hospitalisation, stroke, and death [10,11]. The use of benzodiazepines has also been linked to adverse clinical outcomes, such as falls, increased risk of pneumonia, and dementia [16,17,18].

Recent studies examining residents’ exposure to PIMs in the Australian RACF setting have reported high prevalence of PIM prescribing, ranging from 44% to 81% of residents exposed to PIMs [23,24,25,26,27,28]. In international studies, a systematic review found a prevalence ranging from 18.5% to 82.6% [29]. Many pharmacist-led interventions have been trialled in RACFs to improve QUM. Common approaches have included medication reviews and educational programs, with most showing a lack of association between interventions and reduced adverse drug events (ADEs) [30,31]. A recent systematic review examining pharmacist-led interventions concluded that targeted and tailored interventions are required to improve QUM in RACF settings [30]. Another systematic review emphasised the importance of targeting interventions to those residents who are most at risk of exposure to medications that potentially may cause harm [31].

Several resident and clinical factors have been associated with the increased odds of PIMs in RACFs, including polypharmacy, younger age, and certain medical conditions, such as diabetes and depression [4,29,32]. However, the association of system level factors, such as facility size and staffing arrangements, with the presence of PIMs has been explored less [32]. Associations between facility size and PIMs prevalence have been mixed. A study investigated medication appropriateness in aged care residents using Beers^®^ criteria and found that larger aged care facilities had increased PIMs use [33], while another study showed a smaller RACF size was linked with increased PIMs use [34]. A study explored the association of skilled staff with the use of PIMs, and found that a lower registered nurse (RN) to resident ratio was a predictor of increased PIMs use [33]. A better understanding of PIMs use and associated resident, system and clinical factors may help develop targeted interventions aimed at residents and RACFs most affected by increased number of PIMs.

The objective of this study was to investigate the prevalence of PIMs prescribing and other relevant QUM indicators in RACFs, including the use of antipsychotics and benzodiazepines, utilising baseline data from the PiRACF study. Moreover, we aimed to identify resident, clinical, and system-level factors associated with the use of PIMs.

## 2. Materials and Methods

This study was a cross-sectional analysis of the baseline data from the 15 RACFs particiaptaing in the PiRACF study [35]. The PiRACF study is a cluster randomised controlled trial to evaluate the effectiveness of on-site pharmacists integrated into RACFs care teams to improve medication management. Only residents who are permanent residents of the RACF and over the age of 65 years were included in the study. Data collected included demographic details of residents, medical diagnoses, and medication schedules. A Microsoft Access^®^ (version 16; Microsoft Inc, Seattle, WA, USA) database was designed to capture and store data. Residents’ medications were entered according to the Anatomical Therapeutic Chemical (ATC) classification system [36]. Residents’ data, such as demographics, medications, and medical conditions, were collected at baseline. Other data related to the RACFs, such as number of beds, number of residents, and number of RNs, were collected through surveys completed by the RACF managers.

### 2.1. Data Analysis and Identification of QUM Indicators

Resident’s medications were examined for PIMs using the Beers^®^ 2019 criteria [21]. The Beers^®^ criteria were slightly modified to fit the Australian setting by including medications from the same classes that are available in Australia; this approach was employed in a previous similar Australian study [28]. Use of antipsychotics and benzodiazepines were also analysed. Residents on an antipsychotic with a documented history of major psychiatric illness (schizophrenia and bipolar disorder) have been excluded, as well as residents taking a benzodiazepine and having a history of epilepsy.

### 2.2. Selection of Factors

The selection of factors to test for association with PIMs use was based on a literature review and discussions amongst the study team. Resident’s factors included age, sex, Aboriginal and Torres Strait Islander status, length of stay in the facility, and speaking a language other than English. Clinical factors included total number of medications (polypharmacy), number of chronic medical conditions, Charlson Comorbidity Index (CCI) [37], presence of dementia, and history of falls as per RACF records. System factors included each facility’s bed capacity and RN-to-resident ratio.

Polypharmacy has been associated with numerous negative consequences in the elderly population, such as increased risk of ADEs, drug-to-drug interactions, and cognitive decline [38]. Total number of medications include regular and as pro re nata or PRN (as required use) medications as charted in the RACFs records. The number of medical conditions can also be a risk factor influencing polypharmacy and the number of PIMs [39]. RN-to-resident ratio has been used as a measure of RN staffing [33], which may affect overall quality of care and QUM in RACFs. RN-to-resident ratio was calculated based on the number of registered nurses that work each week divided by the number of residents, as reported by the facility. This was categorised into three categories for analysis, namely, RN-to-residents ratio 1:7 or higher, RN-to-residents ratio between 1:8 to 1:11, and RN-to-residents ratio of 1:12 or lower.

### 2.3. Statistical Analysis

Data were exported from the Microsoft Access^®^ database into SPSS (version 27.0; IBM Corp. Armonk, NY, USA) for statistical analysis. Descriptive statistics included the mean, median and standard deviation for numeric variables and proportion for categorical variables.

The key PIM outcome variable (number of PIMs prescribed for each patient) is a discrete count outcome and therefore regression modelling approaches appropriate for count data were used. To account for the overdispersion that characterise count outcome data, a negative binomial regression was performed, instead of the usual Poisson regression. First, a bivariate analysis was conducted to examine the association between PIM outcome and each covariate. Second, covariates for which an association with the PIM outcome was found in the bivariate model at the level of significance of *p*-value < 0.1 were included as candidates for the final multivariable model. The model also controlled for sex and number of medications. Multicollinearity was tested using the variance inflation factor (VIF). Covariates for which VIF < 6 were kept in the model. For the final model, the level of significance was set at 5%. Any observed result with associated probability value less than 5% (*p*-value < 0.05) was considered statistically significant for all analyses.

## 3. Results

A total of 1357 residents from the 15 participating RACFs were included. The median age was 86 years, 65.1% were female, and 13.3% spoke a language other than English. The median total number of medications used by each resident was 12. The median CCI score was 2, and 53% of residents from the cohort had a diagnosis of dementia. The demographics and clinical characteristics of residents are summarised in Table 1.

Most residents (75.5%) were prescribed at least one PIM, as identified by the Beers Criteria^©^ (Table 2). At least one PIM was charted as regular in 68.1% of the residents, while 34.7% of residents had at least one PIM charted as pro re nata or PRN (as required use). Over 20% of all residents were taking at least one antipsychotic (20.2%) or benzodiazepine/benzodiazepine-like medication (20.9%).

The most common class of medications implicated as PIMs was proton pump inhibitors, comprising 21% of total PIMs, followed by opioids (17.3%). Benzodiazepines and antipsychotics comprised of 27.2% of the total number of PIMs identified (Table 3).

In the final multivariable model (Table 4), five factors showed statistically significant associations with the number of PIMs, namely, age, CCI, history of falls, diagnosis of dementia and RN-to-resident ratio.

Residents with higher CCI score, history of falls, or those who live in facilities with a low RN-to-residents ratio (1:8 or lower) were associated with increased number of PIMs. Residents with a CCI of 3 or more were 1.2 times more likely to have higher PIMs compared to residents with lower CCI scores. Residents with a history of falls were 1.4 times more likely to have more PIMs. Residents with lower RN to resident’s ratio were likely to have an increased number of PIMs.

Older age (>85) and dementia diagnosis were associated with lower number of PIMs. Additionally, residents with dementia were less likely to have PIMs. Amongst all the factors, the strongest association with increased number of PIMs, was history of falls followed by RN-to-resident ratio.

## 4. Discussion

This study examined key QUM indicators in RACFs, including the prevalence rates of PIMs, and prescribing of antipsychotics and benzodiazepines. The study reported the most frequent classes of PIMs prescribed in RACFs and explored the associations between PIM use and various resident and RACF system-level factors. The proportion of residents with one or more PIMs according to the Beers criteria^®^ was 75.5%. The study found a positive association between prescribing PIMs and certain resident factors such as having a history of falls and an increased CCI, while a negative association was found with the presence of dementia diagnosis. The only system factor that was found to be associated with PIMs use was the RN-to-resident ratio, where a lower ratio of RN-to-resident (understaffing) was associated with increased number of PIM prescribing.

The proportion of residents taking at least one regular PIM was 68.1%, with 34% taking at least one PRN PIM. This is consistent with the higher end of the range reported in previous Australian studies [23,24,25,26,27,28]. A systematic review of 21 studies showed a median of 45.5% of residents were prescribed at least one PIM [29]. Internationally, the prevalence of PIMs in RACFs varied depending on what criteria was used and the regions studies were conducted. The prevalence of PIMs was reported higher in Europe than in North America [4]. Interestingly previous studies in Australian RACFs setting have not reported a breakdown of regular and PRN PIMs of residents. This is important as one recent study examined the frequency of administration of PRN relative to regular medications in 8 RACFs found PRN administrations over a 7-day period comprised less than 1% of all administrations [40]. This shows the contribution of PRN medications in RACFs is likely to be small and more attention should be focused on reducing regular PIMs which is evidently high as shown in this study.

The prevalence of residents prescribed regular antipsychotics and regular benzodiazepines in this study was 16.9% and 11.1%, respectively. Antipsychotics are commonly prescribed in RACFs to treat behavioural and psychological symptoms of dementia [41], but their use has been associated with increased risk of falls, stroke and death [42]. The use of benzodiazepines is also commonly used for insomnia, anxiety, and agitation but similarly their use has been associated with falls, increased rates of pneumonia, and increased risk of dementia [16,17,18]. Previous studies have reported higher prevalence of regular antipsychotic for residents in residential aged care settings. Westaway et al., reviewed sixteen studies between 2000 and 2017 and found 13% to 42% of residents, with an average of 26% of residents were prescribed an antipsychotic in RACFs [43]. A study in 2018 found regular benzodiazepines were prescribed in 22.2% of residents [44]. In contrast, our study showed a notable reduction in the use of benzodiazepines and antipsychotics. There has been increased awareness about the use of chemical restraint among RACF residents. One of catalysts for this was the Australian Royal Commission’s enquiry into Aged Care Quality and Safety which emphasized high prevalence of psychotropic use for behavioural and psychological symptoms of dementia in their interim report [45]. This also may be explained by the multiple public health campaigns and interventions aimed at reducing the use of antipsychotics and benzodiazepines in RACFs such as the Reducing the Use of Sedatives (RedUSe) project and the Halting Antipsychotic use in Long Term care (HALT) study as well as the introduction of the NPS Medicinewise dementia education program in Australia [44,46,47].

The most frequently used class of PIMs found in this study was proton pump inhibitors (PPIs), exceeding the use of antipsychotics and benzodiazepines. The long-term use of PPIs has been linked with increased rates of Clostridium difficile infections, pneumonia, fractures, hypomagnesemia and both acute and chronic kidney disease [48,49]. Increasing levels of long-term PPI use over the past two decades have been well documented in Australian studies, partially attributed to changing prescribing patterns [49,50]. This increase is also shown in similar international studies [51,52]. While the use of PPIs is often justified, such as its use in conjunction with NSAIDs or anticoagulants, there are signs of non-evidence-based use of PPIs. A study of RACFs residents in the US found almost half of residents used PPIs for non-evidence-based indications [53]. Due to the safety concerns of long-term PPI use, there may be a need to tailor interventions to review and adjust the duration of PPI when appropriate. Pharmacists may be able to play a key role in assisting medical practitioners to optimise PPI use by implementing regular audits and assessing the need for continuation of PPIs on regular basis in RACF residents.

History of falls was associated with risk of PIMs. This may be explained by residents’ use of antipsychotics, benzodiazepines and hypnotics which have been linked with increased number of falls [54,55]. An association was found between PIMs prescribing and a higher CCI in this cohort. CCI predicts the ten-year mortality for a patient who may have a range of comorbid conditions, however, this association was small however and needs to be interpreted with caution.

This study also found the use of PIMs was inversely associated with the presence of dementia diagnosis. Other studies also found inverse relationship with dementia and PIMs use [56,57,58], while most other studies did not find an association between dementia and PIMs use [33,59,60]. The association between use of PIMs and dementia diagnosis is conflicting and needs further research to determine which medications are more likely to be associated with dementia diagnosis. The inverse association with dementia may be explained by medical practitioners’ and pharmacists’ focus on deprescribing in residents with dementia due to changes in goals of therapy as well as avoiding inappropriate medication that may exacerbate the deterioration of residents’ conditions

A lower RN-to-resident ratio was associated with a higher number of PIMs in this cohort. This finding is consistent with a previous study which found that residents in facilities staffed with fewer RNs relative to the number of residents were at twice the risk of receiving PIMs [33]. A low RN-to-resident ratio maybe a proxy for other quality related factors on the facility level, therefore further investigation is required to better understand the nature of the association between RACF staffing and quality of prescribing. Lower quality of care in RACFs has partly been attributed to inadequate level of nurse staffing [61]. Additionally, a recent review of factors influencing medication safety found that a higher skilled staff number played an important role in preventing medication errors [62]. The shortage of RNs in RACFs has been raised as a concern given its potential impact on delivering quality care, including medication use in RACFs [63,64]. Currently, there is no standard requirement to employ RNs per specific number of residents in RACFs. Due to the complex nature of medication management, the availability of more highly trained staff such as RNs or pharmacists may help reduce medication-related problems, but further evidence is needed [65].

This study shows there is a high level of PIMs use amongst residents of RACFs in Australia. There needs to be a concerted effort to conduct high quality studies examining novel interventions to improve QUM and target those residents most at risk. Implementing integrated pharmacist services in RACFs may help in this endeavour. An example in Australia is the pharmacist in residential aged care facilities study (the PiRACF study) which is a cluster randomised trial that aims to evaluate the effectiveness of embedding a pharmacist within the multidisciplinary team in the aged care facility to improve quality use of medicines [35].

There are some limitations to our study. This is a cross-sectional study; therefore, only the association between examined factors and PIMs can be determined, and there was no scope to assess causality. An implicit limitation of the Beers Criteria is to not take individual’s circumstances into account; therefore, PIM use may be clinically appropriate in some residents. Additionally, all recruited RACFs were from the Australian Capital Territory in Australia and, therefore, generalisability to other regions may be limited.

## 5. Conclusions

Despite recent efforts to improve QUM in RACFs, the extent of PIMs prescribing remains high, with more than two-thirds of residents exposed to at least one regularly used PIM. Long-term PPI use was the most frequent class of PIMs found in this study while a notable reduction in regularly prescribed antipsychotics and benzodiazepines was found compared to previous studies, pointing to a possible change in prescribing patterns. History of falls, younger age and increased CCI scores for residents were found to be associated with an increased number of prescribed PIMs, while facilities with a lower RN-to-resident ratio were also associated with an increased use of PIMs. This study points to a need to further explore factors that might be associated with inappropriate prescribing and tailor interventions targeting those residents most at risk.

## Figures and Tables

**Table 1:** Characteristics of residents.

Variable	N	(%)
**Age (years)**		
65–74	159	11.7
75–84	424	31.2
85 or more	774	57.0
**Sex**		
Male	478	34.9
Female	890	65.1
**Aboriginal and Torres Strait Islander status**		
Yes	6	0.4
No	1362	99.6
**Preferred language**		
English	1186	86.7
Other	182	13.3
**Number of medications**		
Less than 5	131	9.6
5–9	331	22.8
10 or more	922	67.4
**Charlson comorbidity index (CCI)**		
0	178	13.0
1	394	28.8
2	299	21.9
3 or more	497	36.3
**Length of stay (years)**		
Less than 3	911	66.6
3–6	335	24.5
7–12	83	6.1
13 or more	26	1.9
**History of falls**		
Yes	1090	79.7
No	278	20.3
**Dementia diagnosis**		
Yes	725	53.0
No	643	47.0
**Nursing home bed capacity**		
Less than 50	17	1.2
50–100	703	51.4
101–200	648	47.4
**Registered Nurse (RN)-to-resident ratio**		
1:7 or higher	407	29.8
1:8–1:11	557	40.7
1: 12 or lower	404	29.5

**Table 2:** Prevalence of QUM indicators.

Prevalence	Total (N = 1368) (%) *	Regular Use	PRN Use
**Residents with at least one instance of Potentially Inappropriate Medication (PIM)**	1033 (75.5%)	932 (68.1%)	476 (34.7%)
Residents with at least one antipsychotic medication **	275 (20.2%)	230 (16.9%)	99 (7.3%)
Residents with at least one benzodiazepine or benzodiazepine-like medication ***	286 (20.9%)	151 (11.1%)	184 (13.5%)

* The total is not the sum of regular and PRN as sometimes residents were on both simultaneously. ** Residents with a history of major psychiatric illness (schizophrenia and bipolar disorder) have been excluded. *** Residents with a history of epilepsy have been excluded.

**Table 3:** Most commonly prescribed Potentially Inappropriate Medications (PIMs).

Top 10 Most Common PIMs by Drug Class	Total Number of PIMs N = 2734	(%) *
Proton Pump Inhibitors **	**573**	**21**
Pantoprazole	267	9.9
Esomeprazole	140	5.1
Rabeprazole	85	3.1
Opioids ***	**472**	**17.3**
Oxycodone	180	6.6
Buprenorphine	96	3.5
Hydromorphone	58	2.1
**Benzodiazepines**	**373**	**13.6**
Midazolam	123	4.5
Temazepam	99	3.6
Lorazepam	79	2.9
**Antipsychotics**	**373**	**13.6**
Risperidone	134	4.9
Quetiapine	96	3.5
Olanzapine	74	2.7
**Gastrointestinal**	**177**	**6.4**
Metoclopramide	165	6
Prochlorperazine	12	0.43
**Cardiovascular**	**91**	**3.3**
Digoxin	76	2.8
Amiodarone	10	0.4
Diltiazem	2	0.1
**Antiepileptics**	**87**	**3.2**
Pregabalin	56	2.0
Levetiracetam	11	0.4
Phenytoin	6	0.2
**Corticosteroids**	**72**	**2.6**
Prednisolone	46	1.7
Fludrocortisone	7	0.3
Hydrocortisone	7	0.3
**Endocrine**	**51**	**1.9**
Gliclazide	49	1.7
Glipizide	1	0.0
Testosterone	1	0.0
**Antidepressants**	**51**	**1.9**
Amitriptyline	23	0.8
Paroxetine	15	0.5
Nortriptyline	7	0.3

* The percentage of the total number of PIMs (regular and PRN). ** Only proton pump inhibitors with continuous use of 8 weeks or more were included, as indicated by the Beers Criteria^©^. *** Only opioids for residents with history of falls or fractures as indicated by the Beers Criteria©.

**Table 4:** Factors associated with potential inappropriate medications in bivariate and Multivariable model.

Variable	Bivariate Analysis			Multivariable Analysis	
	N	RR (95% CI)	*p* Value *	RR (95% CI)	*p* Value
**Age (years)**			**0.016**		**0.007**
65–74	159	1.00		1.00	
75–84	424	1.022 (0.817–1.277)	0.745	0.971 (0.773–1.219)	0.798
Equal or over 85 years	774	0.837 (0.739–0.944)	0.004	0.784 (0.629–0.976)	0.029
**Sex**			0.235		0.181
Female (0)	890	1.00		1.00	
Male (1)	478	0.946 (0.823–1.086)	0.235	0.906 (0.783–1.047)	0.181
**Aboriginal and Torres Strait Islander status**			0.515		
No	1362	1.00			
Yes	6	1.370 (0.530–3.540)	0.515		
**Preferred Language**			0.420		
English	1186	1.00			
Other	182	0.923 (0.758–1.122)	0.420		
**Number of medications**			0.387		0.655
Fewer than 5	131	1.00		1.00	
5–9	311	1.124 (0.957–1.320)	0.29	1.075 (0.823–1.403)	0.596
10 or more	922	1.176 (1.017–1.360)	0.154	1.113 (0.875–1.416)	0.381
**Charlson comorbidity index (CCI)**			**0.010**		**0.021**
**0**	178	1.00		1.00	0.635
**1**	394	0.864 (0.755–0.990)	0.035	0.944 (0.744–1.198)	0.722
**2**	299	0.972 (0.846–1.117)	0.687	1.046 (0.817–1.339)	0.080
**3 or more**	497	1.149 (1.014–1.303)	0.030	1.222 (0.976–1.530)	
**Number of conditions (subgroups)**			0.545		
Fewer than 5	84	1.00			
5–9	358	1.124 (0.957–1.320)	0.154		
10 or more	922	1.176 (1.017–1.360)	0.029		
**Length of stay**			0.276		
2 years or less	911	1.00			
3–6	335	1.125 (1.034–1.225)	0.006		
7–12	83	1.120 (0.974–1.287)	0.113		
Over than 13 years	26	1.415 (1.105–1.813)	0.006		
**History of falls**			**0.001**		**0.000**
No	1090	1.00		1	
Yes	278	1.448 (1.325–1.582)	0.001	1.445 (1.231–1.696)	0.000
**Dementia diagnosis**			**0.021**		**0.031**
No	643	1.00		1.00	
Yes	725	0.854 (0.790–0.924)	0.001	0.854 (0.740–0.986)	0.031
**Nursing home bed capacity**			0.509		
Fewer than 50 beds	17	1.00			
50–100	703	0.729 (0.536–0.991)	0.044		
101–200	648	0.750 (0.552–0.1021)	0.067		
**Registered Nurse (RN) to resident ratio**			**0.001**		**0.001**
1:7 or higher	404	1.00		1.00	0.060
1:8–1:11	557	1.382 (1.177–1.623)	0.001	1.377 (1.1168–1.623)	0.000
1: 12 or lower	407	1.162 (0.976–1.384)	0.092	1.188 (0.993–1.422)	

* Highlighted in bold if *p*-value of overall factor is <0.05.

## Data Availability

The study dataset will not be made publicly available. Only investigators have access to the trial dataset.
